# Mechanical Damage Modulates Bacterial and Fungal Succession on the Surface of *Hypsizygus marmoreus* During Refrigerated Storage

**DOI:** 10.3390/microorganisms14040762

**Published:** 2026-03-27

**Authors:** Jingming Ma, Mingzheng Zhang, Qian Liu, Xiuling Wang

**Affiliations:** 1 Haide College, Ocean University of China, Qingdao 266100, China; 15610150270@163.com; 2 Marine Science Research Institute of Shandong Province, Qingdao 266104, China; zhangmz0227@163.com; 3 National Engineering Research Center of Edible Fungi, Key Laboratory of Agricultural Genetics and Breeding of Shanghai, Institute of Edible Fungi, Shanghai Academy of Agricultural Sciences, Shanghai 201403, China

**Keywords:** *Hypsizygus marmoreus*, mechanical damage, surface microbiome, microbial succession, postharvest spoilage, food safety

## Abstract

Despite the importance of surface microbiota in postharvest quality, the effects of mechanical damage on microbial succession in *Hypsizygus marmoreus* during refrigerated storage remain insufficiently understood. In this study, 16S rRNA gene and ITS amplicon sequencing were used to characterize the bacterial and fungal communities on intact and mechanically damaged *H. marmoreus* during 15 days of storage at 4 °C. Storage time, rather than mechanical damage, was the main driver of whole-community variation, although mechanical damage accelerated visible spoilage assessed qualitatively. Bacterial communities showed pronounced temporal turnover, shifting from early Firmicutes-rich assemblages to late-stage Proteobacteria-dominated communities, especially *Pseudomonas*. In contrast, fungal communities remained largely dominated by Ascomycota throughout storage, although mechanically damaged mushrooms showed a greater late-stage occurrence of opportunistic yeasts such as *Candida*. Predicted functional and phenotypic analyses further suggested late-stage increases in Gram-negative, aerobic, biofilm-forming, stress-tolerant, and potentially pathogenic bacterial traits. Because these traits were inferred from 16S rRNA gene-based prediction rather than measured directly, they should be interpreted cautiously. Overall, the results suggest that maintaining the physical integrity of *H. marmoreus* during postharvest handling may help preserve quality and delay the emergence of spoilage-associated microbial traits during refrigerated storage.

## 1. Introduction

*Hypsizygus marmoreus* (seafood mushroom) is widely cultivated and valued by consumers for its flavor, nutritional value, and potential health-promoting properties [1]. However, because of its high water content, active metabolism, and lack of a protective cuticle, *H. marmoreus* is highly perishable after harvest [2]. Even under low-temperature storage, the fruiting bodies can rapidly lose quality, showing cap browning, tissue softening, dehydration, and off-odor development [3,4]. Therefore, postharvest spoilage remains a major constraint on the shelf life and marketability of this edible mushroom.

Postharvest deterioration is not driven solely by host physiology; the surface microbiota is also an important determinant of shelf life [5]. Mushroom surfaces harbor complex bacterial and fungal communities that may contribute to tissue degradation and spoilage [6]. Although increasing attention has been given to the postharvest quality of mushrooms, most previous studies have focused mainly on physicochemical changes or microbial composition at single time points [7]. As a result, the temporal succession of bacterial and fungal communities on *H. marmoreus* during refrigerated storage remains insufficiently understood [7,8].

Mechanical damage commonly occurs during harvesting, sorting, packaging, and transport [9]. Such damage can disrupt mushroom tissues and release nutrient-rich cellular exudates, thereby creating favorable microenvironments for microbial attachment and growth [10]. Although mechanical damage is known to accelerate visible decay and physiological senescence [4,9], its effects on microbial community assembly and functional shifts remain poorly understood, especially with respect to the potentially different responses of bacterial and fungal communities [7]. In addition, understanding storage-associated microbial functional shifts, including enhanced biofilm formation or the enrichment of potentially opportunistic taxa, is relevant to postharvest spoilage and microbial food safety [11]. In this context, generalists are taxa that persist across multiple storage stages or conditions, whereas specialists are taxa associated with particular stages or treatments.

To address these gaps, this study investigated the temporal succession of bacterial and fungal communities on the surface of intact (CK) and mechanically damaged (D) *H. marmoreus* during 15 days of refrigerated storage at 4 °C. Using 16S rRNA gene and ITS amplicon sequencing, we aimed to (1) characterize the dynamics and diversity of the surface microbiota; (2) evaluate the potential roles of generalist and specialist taxa in postharvest decay; and (3) examine changes in predicted community functions and phenotypic traits associated with spoilage. This study provides new ecological insight into how mechanical damage modulates the surface microbiota of H. marmoreus and may support the development of targeted strategies to preserve quality and reduce potential microbial safety concerns in fresh edible mushrooms.

## 2. Materials and Methods

### 2.1. Sample Preparation and Experimental Design

**Figure 1:**
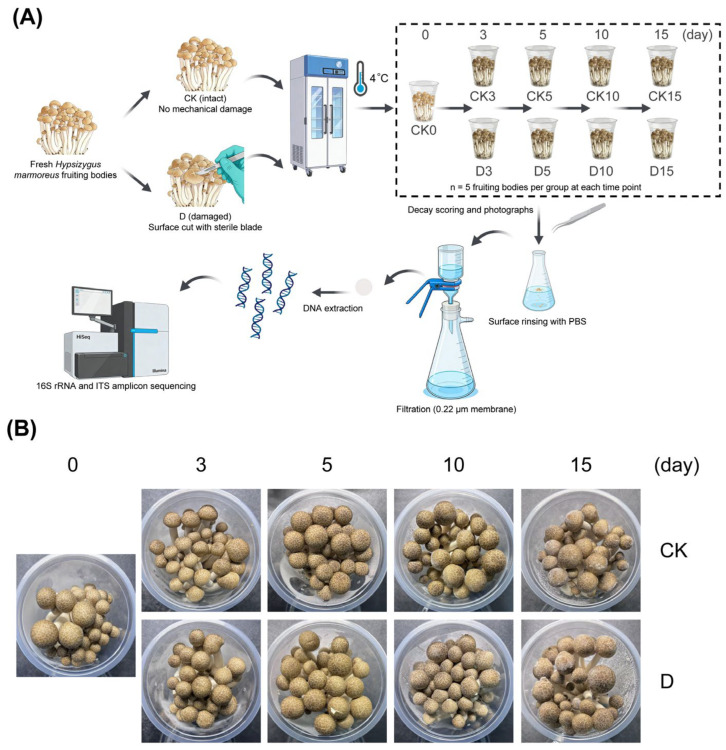
Experimental design, sampling workflow, and representative macroscopic appearance of *Hypsizygus marmoreus* during refrigerated storage. (**A**) Mushrooms were assigned to intact control (CK) and mechanically damaged (D) groups and stored at 4 °C for 15 days. Samples were collected on days 0, 3, 5, 10, and 15 for surface microbiota analysis by 16S rRNA gene and ITS amplicon sequencing. CK0 served as the shared day-0 baseline for the intact and damaged storage trajectories (*n* = 5 biological replicates per group). (**B**) Representative macroscopic appearance of CK and D mushrooms during storage. Visible spoilage features, including cap browning, dehydration, tissue softening, and visible microbial colonization, were documented qualitatively at each sampling point. Figure 1A was created with BioRender.com.

Fresh fruiting bodies of *Hypsizygus marmoreus* were obtained from a commercial cultivation facility in Qingdao, Shandong, China, on the day of harvest and transported to the laboratory. Mushrooms of similar size, with intact caps and stipes and no visible defects, were selected and gently cleaned to remove residual substrate. No antibiotic treatment was applied in this study. Mushrooms were randomly assigned to an intact control group (CK) or a mechanically damaged group (D) (Figure 1A). Mechanical damage was standardized by making a single linear incision (approximately 2–3 mm deep and 0.5–1.5 cm long) on the upper cap surface using a sterile blade. To minimize cross-contamination, the blade was replaced after every five clusters.

All mushrooms were stored at 4 °C in plastic boxes under routine refrigerated storage conditions, with five biological replicates per treatment per sampling point. Samples were collected on days 0, 3, 5, 10, and 15. CK0 served as the shared day-0 baseline for the intact and mechanically damaged trajectories in downstream analyses. In total, 45 samples representing nine storage groups (CK0, CK3, D3, CK5, D5, CK10, D10, CK15, and D15) were included in the microbiome analysis. Macroscopic changes during storage were documented photographically at each sampling point, and visible spoilage features, including cap browning, dehydration, tissue softening, and visible microbial colonization, were recorded as qualitative observations. Relative humidity and gas composition were not independently controlled or instrumentally monitored during storage.

### 2.2. Surface Microbiota Collection and DNA Extraction

Surface-associated microbiota was collected from each biological replicate by immersing the cap and stipe in 0.1 M phosphate buffer (pH 7.4) (Scientific Phygene, Fuzhou, China) and vigorously shaking for approximately 1 min. The resulting wash suspension was filtered through 0.22 μm hydrophilic polycarbonate membranes (Sangon Biotech, Shanghai, China), which were stored at −80 °C until DNA extraction. Total genomic DNA was extracted from the membranes using a modified CTAB-based protocol [12]. DNA quantity and quality were assessed by NanoDrop 2000 spectrophotometry (Thermo Fisher Scientific, Waltham, MA, USA) and 0.8% agarose gel electrophoresis, respectively.

### 2.3. Amplicon Library Preparation and Sequencing

For bacterial community analysis, the V3–V4 region of the 16S rRNA gene was amplified using primers 338F (5′-ACTCCTACGGGAGGCAGCA-3′) and 806R (5′-GGACTACHVGGGTWTCTAAT-3′). For fungal community analysis, the ITS1 region was amplified using primers ITS5 (5′-GGAAGTAAAAGTCGTAACAAGG-3′) and ITS2 (5′-GCTGCGTTCTTCATCGATGC-3′). Sample-specific barcodes were incorporated into the forward primers for multiplex sequencing.

PCR amplification was performed in 25 μL reactions using Q5 High-Fidelity DNA Polymerase (New England Biolabs, Ipswich, MA, USA) according to the sequencing provider’s protocol. For bacterial 16S amplification, thermal cycling consisted of an initial denaturation at 98 °C for 5 min, followed by 25 cycles of 98 °C for 30 s, 52 °C for 30 s, and 72 °C for 45 s, with a final extension at 72 °C for 5 min. For fungal ITS amplification, thermal cycling consisted of an initial denaturation at 98 °C for 5 min, followed by 30 cycles of 98 °C for 30 s, 55 °C for 45 s, and 72 °C for 45 s, with a final extension at 72 °C for 5 min.

Amplicons were checked by 2% agarose gel electrophoresis, purified, quantified, pooled in equimolar amounts, and used for library construction. Libraries were sequenced on the Illumina NovaSeq 6000 platform (Illumina, San Diego, CA, USA) (paired-end 2 × 250 bp) at Shanghai Personal Biotechnology Co., Ltd. (Shanghai, China).

### 2.4. Sequence Processing and Bioinformatics Analysis

Raw demultiplexed reads were processed in QIIME 2 [13] using the q2-dada2 plugin (version 2025.4.0) [14]. After quality filtering, denoising, paired-end merging, and chimera removal, high-quality amplicon sequence variants (ASVs) were obtained. For bacterial 16S ASVs, representative sequences were aligned with MAFFT and used to construct a phylogenetic tree with FastTree2 for phylogeny-based analyses [13,15,16]. Taxonomy was assigned in QIIME 2 using the q2-feature-classifier naïve Bayes classifier, trained on SILVA database (release 138.1) for 16S ASVs and UNITE database (version 9) for ITS ASVs [17,18,19]. ITS ASVs assigned to *H. marmoreus* or other Lyophyllaceae lineages were treated as host-derived and removed from downstream analyses. To account for differences in sequencing depth, ASV tables were rarefied to the minimum post-filtering library size (45,920 reads per sample for 16S and 1352 reads per sample for ITS).

### 2.5. Statistical Analyses

Sequence processing and generation of rarefied ASV tables were conducted through a cloud-based QIIME 2 (version 2025.7) pipeline [13]. All downstream statistical analyses and figure preparation were performed in RStudio (version 2025.05.1) [20] using R (version 4.5.1) [21]. Alpha-diversity indices, including Chao1 richness and Shannon diversity, were calculated from rarefied ASV tables using the vegan package [22]. Between-group differences at corresponding time points and pairwise temporal differences within each storage trajectory were evaluated using two-sided Wilcoxon rank-sum tests.

Beta-diversity patterns were assessed using Bray–Curtis dissimilarities calculated from rarefied ASV tables and visualized by principal coordinates analysis (PCoA). The effects of storage time, treatment, and their interaction on community composition were tested by PERMANOVA using samples collected from days 3 to 15, when both treatments were available.

Generalist and specialist ASVs were identified separately for the intact and mechanically damaged storage trajectories. Generalists were defined as ASVs present in ≥80% of the storage groups within a trajectory and with a mean group-level relative abundance ≥ 0.15%. Specialists were identified using indicator species analysis (IndVal) with permutation testing implemented in the labdsv package [23,24]. Bacterial ASVs with IndVal ≥ 0.80 and fungal ASVs with IndVal ≥ 0.70, both at *p* ≤ 0.05, were classified as specialists, and the group with the highest IndVal was considered the preferred group.

### 2.6. Functional Prediction and Phenotype Inference

Bacterial 16S rRNA ASVs were used to infer predicted functional profiles with PICRUSt2 (v2.3.0) [25], and MetaCyc pathway abundances were obtained [26]. Principal component analysis (PCA) of log1p-transformed pathway relative abundances was conducted using the prcomp function in R. Group-level profiles were used to generate heatmaps of selected MetaCyc pathways, with row-wise Z-score scaling and hierarchical clustering based on Euclidean distance and Ward’s method.

Community phenotypes were inferred with BugBase [27] after normalizing ASV abundances by predicted 16S rRNA gene copy number. Predicted phenotypes included Gram staining status, biofilm-forming potential, pathogenic potential, mobile element content, oxygen utilization, and stress tolerance. These pathway and phenotype profiles were interpreted as predictive inferences based on 16S rRNA gene data rather than direct experimental measurements.

## 3. Results

### 3.1. Overview of Macroscopic Changes and Sequencing Data Summary

During refrigerated storage at 4 °C (Figure 1A), intact control (CK) mushrooms retained visually acceptable quality at the early stage. By days 3–5, moderate cap browning and slight dehydration were observed in both CK and mechanically damaged (D) groups. From day 10 onward, thin mycelial tufts became visible at the cap margins. By day 15, surface dullness, tissue softening, and visible microbial colonization were more evident, particularly in mechanically damaged mushrooms (Figure 1B). These spoilage symptoms were assessed qualitatively based on visual inspection and photographic documentation rather than quantitative measurements.

**Figure 2:**
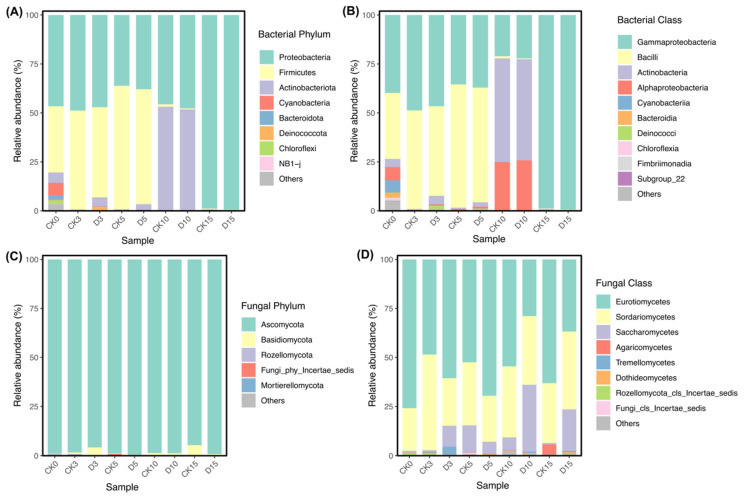
Temporal succession of surface bacterial and fungal communities during refrigerated storage. Relative abundances of major bacterial phyla (**A**) and selected bacterial classes (**B**), and major fungal phyla (**C**) and selected fungal classes (**D**), on the surface of intact control (CK) and mechanically damaged (D) *Hypsizygus marmoreus* stored at 4 °C for 15 days.

The 16S rRNA gene sequencing of surface microbiota yielded 5677 bacterial amplicon sequence variants (ASVs) after quality filtering and bioinformatic preprocessing. In parallel, 669 fungal ASVs were obtained from ITS amplicon sequencing data after removal of host-derived lineages (see Section 2.4). The compositional patterns of the major bacterial and fungal taxa across storage groups are presented in Figure 2.

### 3.2. Temporal Succession of Surface Microbial Communities

At the phylum level, surface bacterial communities were dominated by Proteobacteria (56.4%), Firmicutes (28.3%), and Actinobacteriota (13.3%) (Figure 2A). Clear temporal succession was observed during refrigerated storage. Firmicutes were relatively abundant from day 0 to day 5 but became nearly undetectable by days 10 and 15, whereas Proteobacteria increased progressively and accounted for more than 95% of the bacterial community in both CK15 and D15. Actinobacteriota showed a transient increase at day 10 before declining again by day 15.

These temporal dynamics were also evident at the class level (Figure 2B). Gammaproteobacteria (49.9%) remained dominant throughout storage and largely accounted for the near-monodominance observed at day 15. In contrast, Bacilli (28.2%) were mainly associated with the early storage stage and declined after day 5, whereas Actinobacteria (13.0%) and Alphaproteobacteria (6.6%) showed a transient increase at day 10, suggesting an intermediate restructuring stage of the bacterial community.

Fungal communities showed comparatively less variation at the phylum level and remained overwhelmingly dominated by Ascomycota (98.1%) across storage groups and time points (Figure 2C). At the class level, Eurotiomycetes (54.5%) and Sordariomycetes (32.4%) were the major components of the fungal community, whereas Saccharomycetes became more evident in mechanically damaged mushrooms during late storage (days 10–15) (Figure 2D). Overall, storage time was associated with clear temporal succession in both bacterial and fungal communities, whereas treatment-related differences were more apparent at later storage stages and at lower taxonomic levels.

### 3.3. Alpha and Beta Diversity of Bacterial and Fungal Communities

**Figure 3:**
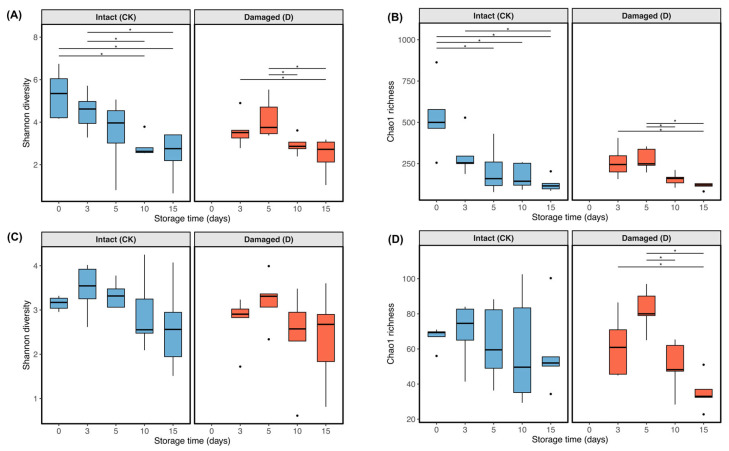
Alpha diversity of surface bacterial and fungal communities during refrigerated storage. Boxplots of Chao1 and Shannon diversity for bacterial communities (**A**,**B**) and fungal communities (**C**,**D**) in intact control (CK) and mechanically damaged (D) *Hypsizygus marmoreus* across storage days 0, 3, 5, 10, and 15. CK0 served as the shared day-0 baseline for both storage trajectories. Brackets indicate significant pairwise differences between time points within each trajectory. Points indicate outliers, and * indicates *p* < 0.05.

Overall, fungal alpha diversity remained lower than bacterial alpha diversity across all storage groups (Figure 3). Bacterial Chao1 richness and Shannon diversity varied over storage time, and the mechanically damaged group showed a modest transient increase at day 5 before both indices declined toward the late stage (days 10–15) (Figure 3A,B). In contrast, fungal alpha diversity showed an initial increase followed by a decline rather than a monotonic trend (Figure 3C,D). The transient maximum occurred at day 3 in the intact control group and at day 5 in the mechanically damaged group, after which both fungal richness and diversity decreased toward the end of storage. However, no significant differences in alpha diversity were detected between CK and D at corresponding sampling points (all *p* > 0.15). Therefore, although the timing of the transient fungal diversity maximum appeared to differ between treatments, this pattern should be interpreted cautiously rather than as a statistically supported effect of mechanical damage.

**Figure 4:**
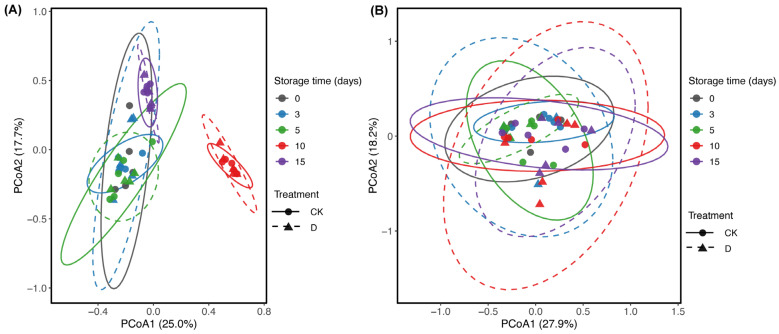
Beta-diversity patterns of surface bacterial and fungal communities during refrigerated storage. Principal coordinates analysis (PCoA) based on Bray–Curtis dissimilarities of bacterial (**A**) and fungal (**B**) communities from intact control (CK) and mechanically damaged (D) *Hypsizygus marmoreus*. Solid and dashed ellipses denote the 95% confidence intervals of the CK and D groups, respectively.

Beta-diversity patterns based on Bray–Curtis dissimilarities are shown in Figure 4. Bacterial communities displayed a clear temporal displacement in ordination space, largely along PCoA1 (25.0%) and PCoA2 (17.7%) (Figure 4A). In contrast, fungal communities showed greater overlap among storage groups, with PCoA1 and PCoA2 explaining 27.9% and 18.2% of the variance, respectively (Figure 4B). PERMANOVA using samples from days 3–15 indicated that storage time explained a substantial proportion of bacterial compositional variation (*R*^2^ = 0.45) but a smaller proportion of fungal variation (*R*^2^ = 0.12). Neither mechanical damage nor the day × treatment interaction had a significant effect on overall community composition for bacteria or fungi (bacteria: treatment, *R*^2^ = 0.018, *p* = 0.248; interaction, *R*^2^ = 0.057, *p* = 0.160; fungi: treatment, *R*^2^ = 0.020, *p* = 0.563; interaction, *R*^2^ = 0.084, *p* = 0.269). Together, these results indicate that storage time, rather than mechanical damage, was the main driver of whole-community beta-diversity patterns.

### 3.4. Specialist and Generalist ASVs Along Intact and Damaged Storage Trajectories

**Figure 5:**
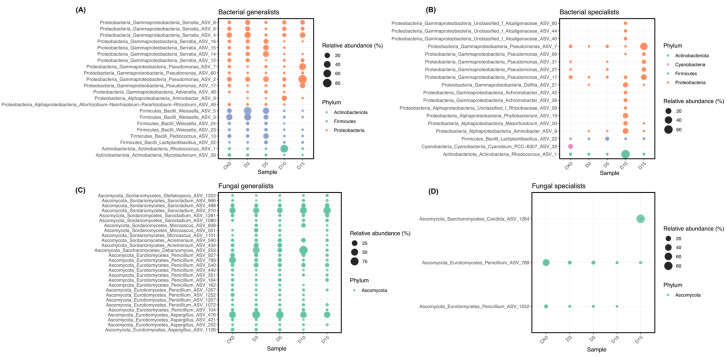
Distribution of generalist and specialist ASVs along the mechanically damaged storage trajectory. Bubble plots show bacterial generalists (**A**), bacterial specialists (**B**), fungal generalists (**C**), and fungal specialists (**D**) identified for the mechanically damaged trajectory using CK0 as the shared day-0 baseline and D3, D5, D10, and D15 as subsequent storage groups. Generalists were defined as ASVs present in ≥80% of storage groups within the trajectory and with a mean group-level relative abundance ≥0.15%, whereas specialists were indicator ASVs significantly associated with particular storage groups, as described in the Methods. Bubble size is proportional to relative abundance, and colors indicate phylum-level taxonomic affiliation. Figure S1 shows the corresponding patterns for the intact storage trajectory.

Generalist and specialist ASVs were analyzed separately for the mechanically damaged storage trajectory (Figure 5). Proteobacteria comprised most bacterial generalists along this trajectory, and several ASVs assigned to *Serratia* and *Pseudomonas* persisted across multiple storage groups, suggesting broad tolerance to refrigerated storage conditions (Figure 5A). In contrast, bacterial specialists showed stronger stage specificity, with the largest number detected at D10 (Figure 5B). Early-stage specialists were mainly Firmicutes, especially *Lactiplantibacillus*-related ASVs, whereas late-stage specialists were dominated by Proteobacteria, including *Achromobacter*, *Delftia*, *Pseudomonas*, and unclassified Alcaligenaceae. A smaller number of Actinobacteriota-associated specialists was also detected at intermediate storage stages. These patterns indicate a progressive replacement of early storage-associated taxa by late-stage Proteobacteria as storage proceeded.

Fungal communities along the mechanically damaged trajectory contained a broad set of generalists belonging mainly to Ascomycota lineages (Figure 5C). Widely distributed ASVs assigned to *Aspergillus*, *Penicillium*, and multiple taxa within Sordariomycetes persisted across storage stages. By contrast, relatively few fungal specialists were identified (Figure 5D), and these were mainly represented by *Penicillium*- and *Candida*-associated ASVs. This pattern suggests that fungal occurrence was dominated by a comparatively stable Ascomycota background, with only a limited subset of taxa showing strong stage specificity. Corresponding distributions of generalists and specialists along the intact storage trajectory are shown in Figure S1.

### 3.5. Predicted Functional and Phenotypic Shifts in Bacterial Communities

**Figure 6:**
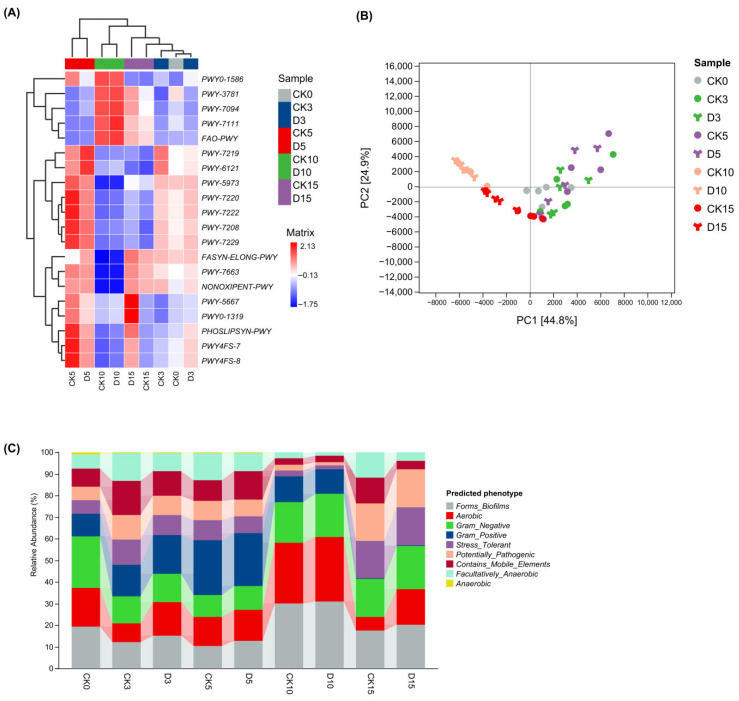
Predicted functional and phenotypic shifts in surface bacterial communities during refrigerated storage. (**A**) Heat map of selected predicted MetaCyc pathways inferred by PICRUSt2. Pathway abundances are shown as row-scaled Z-scores. (**B**) Principal Component Analysis (PCA) of predicted MetaCyc pathway profiles across storage groups. (**C**) Relative contributions of bacterial phenotypes predicted by BugBase after normalization of ASV abundances by predicted 16S rRNA gene copy number.

To examine potential functional changes accompanying bacterial succession during refrigerated storage, we analyzed predicted MetaCyc pathway profiles inferred by PICRUSt2 and predicted community phenotypes inferred by BugBase (Figure 6). Because these approaches infer functions from 16S rRNA gene data rather than measuring them directly, the results should be interpreted as predictive rather than experimentally validated. The PCA of predicted MetaCyc pathway profiles showed clear temporal separation among storage stages, with PC1 and PC2 explaining 44.8% and 24.9% of the total variance, respectively (Figure 6B). Hierarchical clustering of selected pathways further supported this temporal pattern and identified the day-5 samples (CK5 and D5) as a distinct intermediate-stage cluster (Figure 6A).

Representative pathways contributing to the intermediate-stage pattern included NONOXIPENT-PWY (non-oxidative pentose phosphate pathway), PHOSLIPSYN-PWY (phospholipid biosynthesis), and PWY-5667 (CDP-diacylglycerol biosynthesis). By contrast, pathways contributing to the separation of late-stage samples included PWY0-1586 (peptidoglycan maturation) and PWY-3781 (aerobic respiration I, cytochrome *c*). Overall, these temporal differences suggest a predicted restructuring of central metabolism, membrane and cell-envelope related functions, and respiration during storage.

At the phenotype level, predicted community profiles remained relatively similar from day 0 to day 5 (Figure 6C). A clear predicted phenotypic shift emerged at day 10, characterized by an increased relative contribution of bacteria inferred to be Gram-negative, aerobic, and biofilm-forming. By day 15, the predicted phenotypic profiles of both intact and mechanically damaged mushrooms were increasingly characterized by stress-tolerant and potentially pathogenic traits. These phenotype inferences do not demonstrate the direct presence of virulence or pathogenic activity, but they are consistent with the late-stage taxonomic transition toward Proteobacteria-dominated communities described above.

All pathway and phenotype profiles shown here were inferred from 16S rRNA gene data and should be interpreted as predictions rather than direct measurements.

## 4. Discussion

### 4.1. Storage Time Drives Microbial Succession, While Mechanical Damage Accelerates Macroscopic Decay

Postharvest surface microbiota can substantially influence quality deterioration and shelf life [28]. In our study, prolonged refrigerated storage at 4 °C markedly altered the surface microbial assemblages of *H. marmoreus*. Consistent with previous postharvest studies [29,30], storage time was the main driver of whole-community beta-diversity patterns, explaining a much larger proportion of compositional variation than mechanical damage for both bacteria (R^2^ = 0.45) and fungi (R^2^ = 0.12) (Figure 4). However, although mechanical damage did not significantly alter overall beta-diversity trajectories, it clearly accelerated visible spoilage development. The qualitative observations in Figure 1B showed earlier and more pronounced browning, tissue softening, and visible mycelial tufts in mechanically damaged mushrooms, especially at later storage stages. This divergence between whole-community structure and macroscopic decay suggests that physical injury may not shift the entire microbiome uniformly, but can still create localized conditions that favor the rapid establishment of specific spoilage-associated microorganisms [11,31]. By disrupting tissue integrity and increasing the availability of released nutrients, mechanical damage may shorten the effective shelf life of *H. marmoreus* by accelerating the transition from fresh tissue to visible decay.

### 4.2. Temporal Bacterial Succession Toward a Late-Stage Spoilage-Associated Community

The surface bacterial community showed clear temporal succession during refrigerated storage, shifting from an early Firmicutes-rich assemblage to a late-stage Proteobacteria-dominated community (Figure 2A). In particular, Gammaproteobacteria, especially *Pseudomonas*-related taxa, became increasingly dominant toward the end of storage. This pattern is consistent with previous reports showing that many *Pseudomonas* spp. are psychrotrophic and remain metabolically active under refrigeration [32,33,34], where they are frequently associated with tissue deterioration and spoilage development [35,36]. A transient increase in Actinobacteriota and Alphaproteobacteria on day 10 suggests that the bacterial community passed through an intermediate transitional stage before the late-stage Proteobacteria-dominated assemblage became established (Figure 2B). Rather than representing a directly measured mechanistic threshold, this fluctuation is better interpreted as a phase of ongoing community restructuring during storage. By day 15, the near-monodominance of Proteobacteria was accompanied by more evident qualitative deterioration, supporting a close association between bacterial succession and late-stage spoilage development in *H. marmoreus* [35,36].

### 4.3. More Gradual Fungal Succession and the Emergence of Opportunistic Yeasts in Damaged Tissues

Compared with bacteria, fungal communities changed more gradually during refrigerated storage. Ascomycota remained dominant across storage groups (Figure 2C), consistent with reports from other refrigerated postharvest systems [37,38,39]. In our dataset, this relative stability was also reflected by the broader overlap of fungal samples in ordination space and the smaller proportion of compositional variance explained by storage time than in bacteria (Figure 4). However, phylum-level stability did not preclude shifts at lower taxonomic levels. In mechanically damaged mushrooms, Saccharomycetes became more evident during late storage, and *Candida*-associated specialist ASVs were detected in the damaged trajectory (Figure 2D and Figure 5D). One plausible explanation is that tissue disruption created localized wound-associated microenvironments enriched in moisture and soluble nutrients, as mechanical damage during postharvest handling and storage is known to alter mushroom tissue integrity and quality [40,41,42]. These localized changes may have increased ecological opportunities for opportunistic yeasts in damaged tissues during late storage, although this mechanism was not directly measured in the present study. Therefore, this interpretation should be regarded as a plausible ecological explanation rather than a demonstrated mechanism. Overall, the fungal data suggest relatively limited whole-community restructuring during refrigerated storage, but greater ecological opportunity for opportunistic yeasts in mechanically damaged tissues at later storage stages.

### 4.4. Ecological Roles of Generalists and Specialists During Community Assembly

Classifying the surface microbiome into generalist and specialist taxa provided an ecological framework for interpreting community assembly during storage [43,44]. In this study, generalists were defined as ASVs present in ≥80% of storage groups within a trajectory and with mean group-level relative abundance ≥0.15%, whereas specialists were indicator ASVs associated with particular storage groups. This distinction helps separate broadly persistent taxa from stage-associated taxa that may contribute disproportionately to storage-related succession [31,43,44].

In the bacterial community, early-stage specialists were mainly Firmicutes, especially *Lactiplantibacillus*-related ASVs (Figure 5B). As common colonizers of fresh produce, lactic acid bacteria may transiently limit spoilage-associated taxa through rapid resource use, organic acid production, and competitive exclusion. As storage progressed, however, tissue senescence and physical injury likely weakened surface integrity and increased nutrient leakage, reducing the relative advantage of these early colonizers [40,41,42]. In parallel, late-stage specialists were increasingly represented by Proteobacteria, including *Achromobacter*, *Delftia*, and *Pseudomonas* (Figure 5B), taxa commonly associated with browning, softening, and off-odour development in refrigerated mushrooms [35,36]. This replacement suggests a transition from an early community dominated by common surface colonizers to a late-stage assemblage more closely associated with tissue deterioration.

Bacterial generalists, particularly *Serratia* and some *Pseudomonas* ASVs, persisted across storage groups (Figure 5A), indicating broader ecological tolerance under refrigerated conditions. Their persistence may reflect cold adaptation and surface-associated survival strategies, including extracellular polymeric substance production and biofilm formation, although these mechanisms were not directly measured here [32,33,45]. By contrast, fungal communities contained relatively few specialists (Figure 5D) and a broader background of Ascomycota generalists, including *Aspergillus* and *Penicillium* (Figure 5C). Similar gradual fungal temporal dynamics have been reported in other postharvest systems [37,38,39]. Taken together, these patterns suggest that bacterial turnover was more dynamic and stage-dependent than fungal turnover, and therefore more closely associated with postharvest decay progression in *H. marmoreus*.

### 4.5. Predicted Functional Shifts and Implications for Microbial Food Safety

Taxonomic changes during refrigerated storage were accompanied by predicted functional and phenotypic shifts in the bacterial community. Because MetaCyc pathway profiles and BugBase phenotypes were inferred from 16S rRNA gene data rather than measured directly, they should be interpreted as predictions rather than experimentally confirmed functions or traits [25,27]. The temporary increase in several pathways at day 5 (Figure 6A,B) may reflect a short-term adaptive response of the bacterial community to refrigerated storage and early tissue senescence [36,46,47]. By contrast, the phenotype profile shifted more clearly after day 10, with increased predicted contributions from Gram-negative, aerobic, and biofilm-forming bacteria (Figure 6C) [45,48]. This predicted transition was consistent with the late-stage increase in *Pseudomonas* and other Proteobacteria observed at the taxonomic level.

Biofilm formation is widely recognized as a strategy that enhances bacterial adhesion, persistence, and stress tolerance in food-associated environments [45,48,49]. Cold stress has also been reported to promote thicker biofilms and the upregulation of virulence-related proteins in some bacteria [45,50,51]. By day 15, both intact and mechanically damaged mushrooms showed higher predicted contributions of stress-tolerant and potentially pathogenic phenotypes (Figure 6C). However, these phenotype inferences do not demonstrate actual pathogenicity, virulence, or foodborne risk; rather, they indicate a predicted late-stage community profile that may be more persistent and spoilage-associated under refrigeration [27]. Mechanical damage may further accelerate the development of this predicted profile by disrupting tissues and increasing nutrient availability on the mushroom surface [40,41,42].

From a postharvest perspective, these results suggest that maintaining the physical integrity of *H. marmoreus* during handling may help delay not only visible deterioration but also the emergence of spoilage-associated and biofilm-associated bacterial traits during refrigerated storage [40,48].

## 5. Conclusions

In summary, refrigerated storage was the main driver of temporal microbial succession on the surface of *Hypsizygus marmoreus*, whereas mechanical damage accelerated visible spoilage during storage. Bacterial communities showed stronger temporal turnover than fungal communities, shifting from early Firmicutes-rich assemblages to late-stage communities dominated by Proteobacteria, especially *Pseudomonas*. Fungal communities remained largely dominated by Ascomycota, although mechanically damaged mushrooms showed a greater late-stage occurrence of opportunistic yeasts such as *Candida*. Predicted functional and phenotypic analyses further suggested that late storage stages were associated with increased contributions of Gram-negative, aerobic, biofilm-forming, stress-tolerant, and potentially pathogenic bacterial traits. Because these traits were inferred from 16S rRNA gene-based prediction rather than measured directly, they should be interpreted cautiously. Overall, maintaining the physical integrity of *H. marmoreus* during postharvest handling may help preserve quality and delay the emergence of spoilage-associated microbial traits during refrigerated storage.

## Data Availability

The original data presented in the study are openly available in the NCBI Se-quence Read Archive under BioProject accession PRJNA1401682.

## Supplementary Materials

The following supporting information can be downloaded at https://www.mdpi.com/article/10.3390/microorganisms14040762/s1: Figure S1. Distribution of generalist and specialist ASVs along the intact storage trajectory. Bubble plots illustrate bacterial generalists (A) and specialists (B), alongside fungal generalists (C) and specialists (D), identified across the intact control samples at days 0, 3, 5, 10, and 15 (CK0–CK15). Bubble size is proportional to relative abundance, while colors denote phylum-level taxonomic affiliations. This figure serves as a comparative baseline to the mechanically damaged trajectory presented in Figure 5.

## References

[1] Angelini P., Flores G.A., Cusumano G., Venanzoni R., Pellegrino R.M., Zengin G., Di Simone S.C., Menghini L., Ferrante C. (2023). Bioactivity and Metabolomic Profile of Extracts Derived from Mycelial Solid Cultures of *Hypsizygus marmoreus*. Microorganisms.

[2] Xing Z., Wang Y., Feng Z., Tan Q. (2008). Effect of Different Packaging Films on Postharvest Quality and Selected Enzyme Activities of *Hypsizygus marmoreus* Mushrooms. J. Agric. Food Chem..

[3] González-Fandos E., Giménez M., Olarte C., Sanz S., Simón A. (2000). Effect of packaging conditions on the growth of micro-organisms and the quality characteristics of fresh mushrooms (*Agaricus bisporus*) stored at inadequate temperatures. J. Appl. Microbiol..

[4] Zivanovic S., Busher R., Kim K. (2000). Textural Changes in Mushrooms (*Agaricus bisporus*) Associated with Tissue Ultrastructure and Composition. J. Food Sci..

[5] Largeteau M.L., Savoie J.-M. (2010). Microbially induced diseases of *Agaricus bisporus*: Biochemical mechanisms and impact on commercial mushroom production. Appl. Microbiol. Biotechnol..

[6] Sivanesan D. (2003). Diversity Among Bacteria Causing Blotch Disease on the Commercial Mushroom, Agaricus Bisporus /. http://hdl.handle.net/10464/2300.

[7] Zhang K., Pu Y.-Y., Sun D.-W. (2018). Recent advances in quality preservation of postharvest mushrooms (*Agaricus bisporus*): A review. Trends Food Sci. Technol..

[8] Nazah J. (2015). Food Spoilage: Microorganisms and their prevention. Asian J. Plant Sci. Res..

[9] Opara U.L., Pathare P.B. (2014). Bruise damage measurement and analysis of fresh horticultural produce—A review. Postharvest Biol. Technol..

[10] Barth M., Hankinson T.R., Zhuang H., Breidt F., Sperber W.H., Doyle M.P. (2009). Microbiological Spoilage of Fruits and Vegetables. Compendium of the Microbiological Spoilage of Foods and Beverages.

[11] Snyder A.B., Martin N., Wiedmann M. (2024). Microbial food spoilage: Impact, causative agents and control strategies. Nat. Rev. Microbiol..

[12] Porebski S., Bailey L.G., Baum B.R. (1997). Modification of a CTAB DNA extraction protocol for plants containing high polysaccharide and polyphenol components. Plant Mol. Biol. Rep..

[13] Bolyen E., Rideout J.R., Dillon M.R., Bokulich N.A., Abnet C.C., Al-Ghalith G.A., Alexander H., Alm E.J., Arumugam M., Asnicar F. (2019). Reproducible, interactive, scalable and extensible microbiome data science using QIIME 2. Nat. Biotechnol..

[14] Callahan B.J., Mcmurdie P.J., Rosen M.J., Han A.W., Johnson A.J.A., Holmes S.P. (2016). DADA2: High-resolution sample inference from Illumina amplicon data. Nat. Methods.

[15] Katoh K., Standley D.M. (2013). MAFFT Multiple Sequence Alignment Software Version 7: Improvements in Performance and Usability. Mol. Biol. Evol..

[16] Price M.N., Dehal P.S., Arkin A.P. (2010). FastTree 2—Approximately Maximum-Likelihood Trees for Large Alignments. PLoS ONE.

[17] Bokulich N.A., Kaehler B.D., Rideout J.R., Dillon M., Bolyen E., Knight R., Huttley G.A., Gregory Caporaso J. (2018). Optimizing taxonomic classification of marker-gene amplicon sequences with QIIME 2’s q2-feature-classifier plugin. Microbiome.

[18] Quast C., Pruesse E., Yilmaz P., Gerken J., Schweer T., Yarza P., Peplies J., Glöckner F.O. (2013). The SILVA ribosomal RNA gene database project: Improved data processing and web-based tools. Nucleic Acids Res..

[19] Nilsson R.H., Larsson K.-H., Taylor A.F.S., Bengtsson-Palme J., Jeppesen T.S., Schigel D., Kennedy P., Picard K., Glöckner F.O., Tedersoo L. (2019). The UNITE database for molecular identification of fungi: Handling dark taxa and parallel taxonomic classifications. Nucleic Acids Res..

[20] Posit Team (2025). RStudio: Integrated Development Environment for R.

[21] R Core Team (2025). R: A Language and Environment for Statistical Computing.

[22] Oksanen J., Simpson G.L., Blanchet F.G., Kindt R., Legendre P., Minchin P.R., O’Hara R.B., Solymos P., Stevens M.H.H., Szoecs E. (2022). Vegan: Community Ecology Package.

[23] Dufrêne M., Legendre P. (1997). Species assemblages and indicator species: The need for a flexible asymmetrical approach. Ecol. Monogr..

[24] Roberts D.W. (2025). Labdsv: Ordination and Multivariate Analysis for Ecology.

[25] Douglas G.M., Maffei V.J., Zaneveld J.R., Yurgel S.N., Brown J.R., Taylor C.M., Huttenhower C., Langille M.G.I. (2020). PICRUSt2 for prediction of metagenome functions. Nat. Biotechnol..

[26] Caspi R., Billington R., A Fulcher C., Keseler I.M., Kothari A., Krummenacker M., Latendresse M., E Midford P., Ong Q., Ong W.K. (2018). The MetaCyc database of metabolic pathways and enzymes. Nucleic Acids Res..

[27] Ward T., Larson J., Meulemans J., Hillmann B., Lynch J., Sidiropoulos D., Spear J.R., Caporaso G., Blekhman R., Knight R. (2017). BugBase predicts organism-level microbiome phenotypes. bioRxiv.

[28] Lei X., Liu Y., Guo Y., Wang W., Zhang H., Yi L., Zeng K. (2022). Debaryomyces nepalensis reduces fungal decay by affecting the postharvest microbiome during jujube storage. Int. J. Food Microbiol..

[29] Riachy R.A., Strub C., Durand N., Chochois V., Lopez-Lauri F., Fontana A., Schorr-Galindo S. (2024). The influence of long-term storage on the epiphytic microbiome of postharvest apples and on penicillium expansum occurrence and patulin accumulation. Toxins.

[30] Yang C., Yang J., Zhou Y., Ou Y., Wang Z., Qi W., Huang R., Chai S., Yang H., Zhou Y. (2025). Biochemical characterization and bacterial diversity of Agrocybe aegerita during postharvest storage. Front. Microbiol..

[31] Xia F., Zhang C., Jiang Q., Wu Z., Cao S., Wu P., Gao Y., Cheng X. (2023). Microbiome analysis and growth behaviors prediction of potential spoilage bacteria inhabiting harvested edible mushrooms. J. Plant Dis. Prot..

[32] Abraham W.P., Raghunandanan S., Gopinath V., Suryaletha K., Thomas S.K. (2020). Deciphering the Cold Adaptive Mechanisms in Pseudomonas psychrophila MTCC12324 Isolated from the Arctic at 79° N. Curr. Microbiol..

[33] Bao C., Li M., Zhao X., Shi J., Liu Y., Zhang N., Zhou Y., Ma J., Chen G., Zhang S. (2023). Mining of key genes for cold adaptation from Pseudomonas fragi D12 and analysis of its cold-adaptation mechanism. Front. Microbiol..

[34] Xiong L., Li Y., Yu H., Wei Y., Li H., Ji X. (2023). Whole genome analysis and cold adaptation strategies of Pseudomonas sivasensis W-6 isolated from the Napahai plateau wetland. Sci. Rep..

[35] Qiu W., Huang Y., Zhao C., Lin Z., Lin W., Wang Z. (2019). Microflora of fresh white button mushrooms (*Agaricus bisporus*) during cold storage revealed by high-throughput sequencing and MALDI-TOF mass spectrometry fingerprinting. J. Sci. Food Agric..

[36] Hou F., Yi F., Song L., Zhan S., Zhang R., Han X., Sun X., Liu Z. (2023). Bacterial community dynamics and metabolic functions prediction in white button mushroom (*Agaricus bisporus*) during storage. Food Res. Int..

[37] Shen Y., Nie J., Dong Y., Kuang L., Li Y., Zhang J. (2018). Compositional shifts in the surface fungal communities of apple fruits during cold storage. Postharvest Biol. Technol..

[38] Li M., Yang S., Peng L., Zeng K., Feng B., Jingjing Y. (2022). Compositional shifts in fungal community of chestnuts during storage and their correlation with fruit quality. Postharvest Biol. Technol..

[39] Zhao L., Li H., Liu Z., Hu L., Xu D., Zhu X., Mo H. (2024). Quality changes and fungal microbiota dynamics in stored jujube fruits: Insights from high-throughput sequencing for food preservation. Foods.

[40] Walkowiak-Tomczak D., Idaszewska N., Bieńczak K., Kómoch W. (2020). The effect of mechanical actions occurring during transport on physicochemical changes in agaricus bisporus mushrooms. Sustainability.

[41] Guan L., Hao S., Chen Y., Yang M., Guo Y., Suguro R., Abbas A. (2025). CaCl_2_ retards mechanical damage induced by simulated-transport vibration and promotes mushroom (agaricus bisporus) quality during storage. Food Biophys..

[42] Wang Z., Ji N., Yang M., Chen Y., Guo Y., Suguro R. (2025). Mechanism of polyethylene glycol on reducing mechanical damage in white mushrooms during simulated logistics vibrations and storage. Postharvest Biol. Technol..

[43] Ren Y., Ge W., Dong C., Wang H., Zhao S., Li C., Xu J., Liang Z., Han Y. (2023). Specialist species of fungi and bacteria are more important than the intermediate and generalist species in near-urban agricultural soils. Appl. Soil Ecol..

[44] Niu X., Sun X., Bai Y., Wen N., Wei X., Wei G., Shu D. (2025). Contrasting the relative importance of microbial generalists and specialists in maintaining assembly processes and community stability. Environ. Microbiol..

[45] Liu J., Wu S., Feng L., Wu Y., Zhu J. (2023). Extracellular matrix affects mature biofilm and stress resistance of psychrotrophic spoilage Pseudomonas at cold temperature. Food Microbiol..

[46] Jiang K., Li L., Yang Z., Chen H., Qin Y., Brennan C. (2023). Variable characteristics of microbial communities and volatile organic compounds during post-harvest storage of wild morel mushrooms. Postharvest Biol. Technol..

[47] Liu Y., Brennan C., Jiang K., Li L., Qin Y., Chen H. (2023). Quality and microbial community changes in three kinds of Boletus wild mushroom during cold storage. Postharvest Biol. Technol..

[48] Fanelli F., Caputo L., Quintieri L. (2021). Phenotypic and genomic characterization of Pseudomonas putida ITEM 17297 spoiler of fresh vegetables: Focus on biofilm and antibiotic resistance interaction. Curr. Res. Food Sci..

[49] Abebe G. (2020). The role of bacterial biofilm in antibiotic resistance and food contamination. Int. J. Microbiol..

[50] Yan J., Xie J. (2020). Comparative proteome analysis of shewanella putrefaciens WS13 mature biofilm under cold stress. Front. Microbiol..

[51] Quintieri L., Fanelli F., Zühlke D., Caputo L., Logrieco A.F., Albrecht D., Riedel K. (2020). Biofilm and pathogenesis-related proteins in the foodborne P. fluorescens ITEM 17298 with distinctive phenotypes during cold storage. Front. Microbiol..

