# Supplementary material for: Mechanical Damage Modulates Bacterial and Fungal Succession on the Surface of Hypsizygus marmoreus During Refrigerated Storage
Source: Microorganisms. 2026 Mar 27;14(4):762. doi: 10.3390/microorganisms14040762 (PMC13119192; doi:10.3390/microorganisms14040762)
Supplement: Supplementary file 1 [file microorganisms-14-00762-s001.zip › microorganisms-4218458-supplementary.pdf]

Mechanical Damage Modulates Surface Bacterial and Fungal Succession on *Hypsizygus marmoreus* Surfaces During Refrigerated Storage

Jingming Ma [1], Mingzheng Zhang [2], Qian Liu [2*], and Xiuling Wang [3*]
1 Haide College, Ocean University of China, Qingdao 266100, Shandong, China
2 Marine Science Research Institute of Shandong Province, Qingdao 266104, Shandong, China
3 National Engineering Research Center of Edible Fungi, Key Laboratory of Agricultural Genetics and Breeding of Shanghai, Institute of Edible Fungi, Shanghai Academy of Agricultural Sciences, Shanghai 201403, China

Correspondence*: Qian Liu (raulliuqian@163.com)
Xiuling Wang (wang.xiuling@outlook.com)

Supplementary Figures

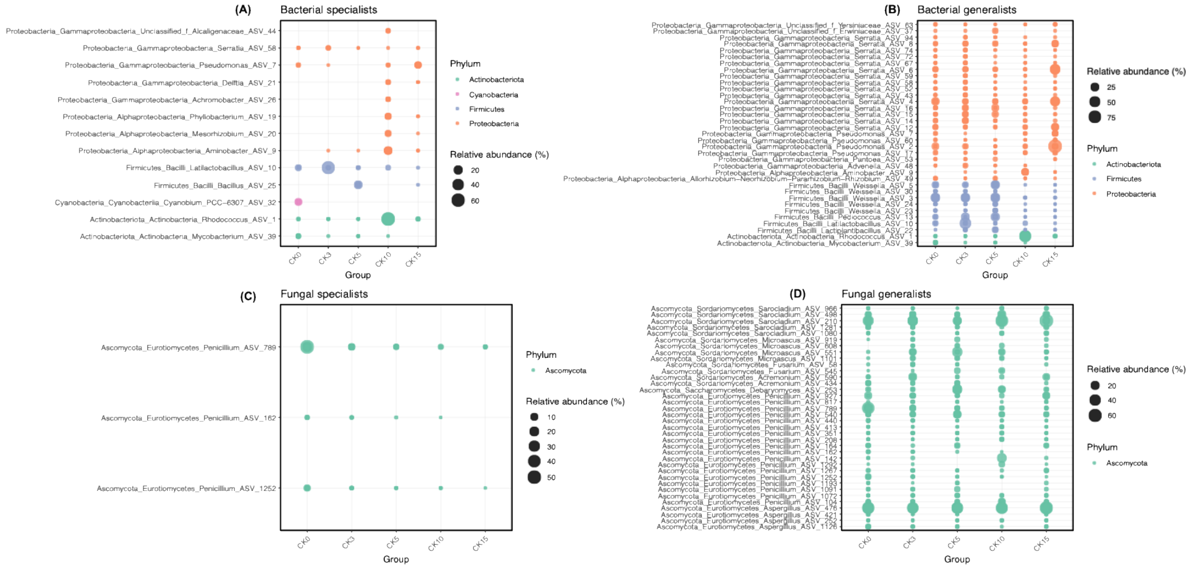

Figure S1. Distribution of generalist and specialist ASVs along the intact storage trajectory. Bubble plots show bacterial generalists (A), bacterial specialists (B), fungal generalists (C), and fungal specialists (D) identified for the intact storage trajectory across CK0, CK3, CK5, CK10, and CK15. Generalists were defined as ASVs present in ≥ 80% of storage groups within the trajectory and with a mean group-level relative abundance ≥ 0.15%, whereas specialists were indicator ASVs significantly associated with particular storage groups, as described in the Methods. Bubble size is proportional to relative abundance, and colors indicate phylum-level taxonomic affiliation.
